# The ultraviolet B inflammation model: Postinflammatory hyperpigmentation and validation of a reduced UVB exposure paradigm for inducing hyperalgesia in healthy subjects

**DOI:** 10.1002/ejp.1353

**Published:** 2019-02-01

**Authors:** Pieter S. Siebenga, Guido van Amerongen, Erica S. Klaassen, Marieke L. de Kam, Robert Rissmann, Geert Jan Groeneveld

**Affiliations:** ^1^ Centre for Human Drug Research Leiden The Netherlands; ^2^ Leiden University Medical Centre Leiden The Netherlands; ^3^ Leiden Academic Centre for Drug Research Leiden The Netherlands

## Abstract

**Background:**

Pain models are commonly used in drug development to demonstrate analgesic activity in healthy subjects and should therefore not cause long‐term adverse effects. The ultraviolet B (UVB) model is a model for inflammatory pain in which three times the minimal erythema dose (3MED) is typically applied to induce sensitization. Based on reports of long‐lasting postinflammatory hyperpigmentation (PIH) associated with 3MED, it was decided to investigate the prevalence of PIH among subjects who were previously exposed to 3MED at our research centre. In addition, re‐evaluation of the UVB inflammation model using a reduced exposure paradigm (2MED) was performed in healthy subjects.

**Methods:**

In the first study, all 142 subjects previously exposed to 3MED UVB were invited for a clinical evaluation of PIH. In the second study, 18 healthy subjects were exposed to 2MED UVB, and heat pain detection threshold (PDT) and PIH were evaluated.

**Results:**

In total, 78 of the 142 subjects responded. The prevalence of PIH among responders was 53.8%. In the second study, we found a significant and stable difference in PDT between UVB‐exposed and control skin 3 hr after irradiation; 13 hr post‐irradiation, the least squares mean estimate of the difference in PDT ranged from −2.6°C to −4.5°C (*p *<* *0.0001). Finally, the prevalence of PIH was lower in the 2MED group compared to the 3MED group.

**Conclusions:**

The 3MED model is associated with a relatively high prevalence of long‐lasting PIH. In contrast, 2MED exposure produces stable hyperalgesia and has a lower risk of PIH and is therefore recommended for modelling inflammatory pain.

**Significance:**

Postinflammatory hyperpigmentation is an unwanted long‐term side effect associated with the UVB inflammation model using the 3× minimal erythema dose (3MED) paradigm. In contrast, using a 2MED paradigm results in hyperalgesia that is stable for 36 hr and has a lower risk of inducing postinflammatory hyperpigmentation.

## 1 INTRODUCTION

Evoked pain models in human subjects are commonly used in the early stages of clinical drug development for demonstrating analgesic activity and determining the compound's active dose. Ideally, a pain model should be easy to perform and should provide reproducible, reliable results under standardized conditions, but it must not cause tissue damage or have long‐term adverse side effects.

The ultraviolet B (UVB) pain model is a commonly used model for studying inflammatory pain, as its effects are sensitive to anti‐inflammatory analgesics, including non‐steroidal anti‐inflammatory drugs (NSAIDs) (Staahl & Drewes, 2004; van Amerongen, de Boer, Groeneveld, & Hay, 2016). This model consists of exposing a patch of skin to UVB irradiation, which leads to a localized reduction in the heat pain threshold due to inflammation; this phenomenon is known as heat allodynia. Typically, three times the minimal erythema dose (3MED) is used to induce hyperalgesia (Andresen et al., 2011; Gustorff, Anzenhofer, Sycha, Lehr, & Kress, 2004; Gustorff, Hauer, Thaler, Seis, & Draxler, 2011; Gustorff et al., 2013; Harrison, Young, & McMahon, 2004; Lo Vecchio, Petersen, & Finocchietti, 2015; Loudon et al., 2018; Okkerse et al., 2017; Sycha et al., 2003), although some groups reported the use of 1x (1MED) and 2x (2MED) the minimal erythema dose (Bauer et al., 2015; Ing Lorenzini et al., 2011, 2012).

**Figure 1:**
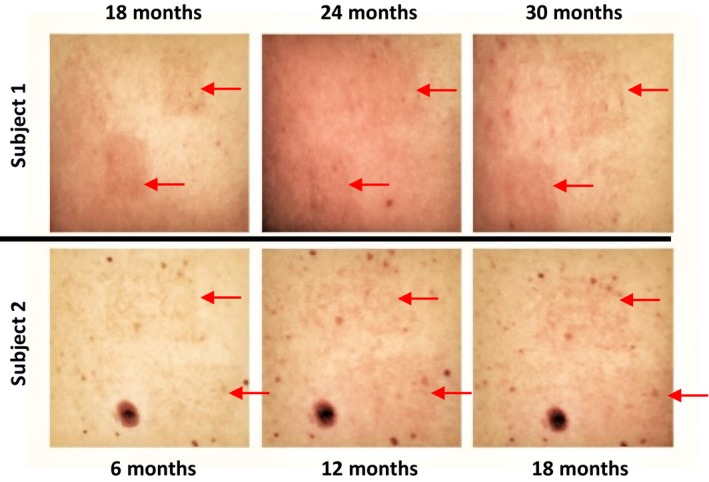
Example images of two subjects in the observational study with postinflammatory hyperpigmentation at the irradiated areas (arrows). The images in the top row were taken 18, 24 and 30 months after UVB irradiation in one subject. The images in the bottom row were taken 6, 12 and 18 months after UVB irradiation in a different subject

From 2012 to 2015, our research centre applied the 3MED UVB model in six studies involving a total of 142 subjects. Beginning in 2015 onwards, some subjects started to report hyperpigmentation of the area of skin that was exposed to UVB, which lasted longer than expected (Figure 1). This postinflammatory hyperpigmentation (PIH) is an acquired form of hypermelanosis that can occur after skin inflammation and/or injury. Although PIH can occur in all skin types, it is generally more common among individuals with skin of colour, including African Americans, Hispanics/Latinos, Asians, Native Americans, Pacific Islanders and persons of Middle Eastern descent (Davis & Callender, 2010). A wide range of aetiologies has been described, including skin diseases (e.g., acne vulgaris, atopic dermatitis, impetigo, plaque psoriasis and lichen planus); bacterial, fungal and viral infections; allergic reactions; medication‐induced PIH; and cutaneous injuries as a result of topical irritants, sunburns and other types of burns, and cosmetic procedures (Taylor, Grimes, Lim, Im, & Lui, 2009).

Hyperpigmentation results from either melanin overproduction or the irregular dispersion of melanin (i.e., pigmentary incontinence) following cutaneous inflammation (Davis & Callender, 2010). Pigmentary incontinence results from the destruction of the basal cell layer (Masu & Seiji, 1983), which allows macrophages to accumulate in the upper dermis, where they phagocytize degenerating basal keratinocytes and melanocytes. The release of melanin from these melanocytes is believed to result in hyperpigmentation (Taylor et al., 2009). Although occasional exposure to sunlight has been associated with a reduced risk of skin cancer (Kennedy, Bajdik, Willemze, De Gruijl, & Bouwes Bavinck, 2003), cumulative exposure to solar UV radiation—particularly UVB radiation—is a major risk factor for developing basal cell carcinoma, squamous cell carcinoma, melanoma and cataract, and should therefore not be taken lightly (Armstrong & Kricker, 2001; Armstrong et al., 2009; Cruickshanks, Klein, & Klein, 1992; Hodge, Whitcher, & Satariano, 1995).

Based on our finding that the 3MED UVB model appeared to be associated with long‐term PIH in some patients, it was decided to perform two studies. The aim of the first study was to measure the prevalence of PIH among all subjects who were previously exposed to the 3MED UVB inflammation model at our centre. The second study was designed to evaluate the short‐term tolerability and efficacy of inflammation using two times the minimal erythema dose (2MED) in order to test whether this lower amount of UVB exposure is associated with a lower frequency and/or severity of PIH in healthy subjects.

## 2 METHODS

Both studies were conducted at the Clinical Research Unit of the Centre for Human Drug Research (CHDR) in Leiden, the Netherlands. Both studies were conducted in accordance with the Declaration of Helsinki and its amendments and in accordance with the Guidelines for Good Clinical Practice. Both protocols were approved by the Medical Ethics Committee approval prior to their initiation; Study I was approved by the Medical Ethics Research Committee of Leiden University Medical Centre (Leiden, the Netherlands), and Study II was approved by the Foundation BEBO (*Stichting Beoordeling Ethiek Biomedisch Onderzoek*) in Assen, the Netherlands. The studies were registered under ToetsingOnline number NL60563.058.17 (Study I) and NL63598.056.17 (Study II).

### 2.1 Study I

#### 2.1.1 Study design

In this observational study, all 142 subjects who previously participated in the studies CHDR0729, CHDR1311, CHDR1422, CHDR1425, CHDR1431 and CHDR1440 (Hay, Okkerse, van Amerongen, & Groeneveld, 2016; Loudon et al., 2018; Okkerse et al., 2017; van Amerongen, Siebenga, de Kam, Hay, & Groeneveld, 2018) and were exposed to 3MED UVB irradiation were contacted and invited to visit our clinical research unit in order to evaluation the area(s) that were exposed to UVB irradiation. In order to maximize the number of respondents, the subjects were given the option to complete the questionnaire (see below) at home and provide a self‐made photograph of the skin.

All participants provided written informed consent. The evaluation included a medical interview, a physical examination of the exposed area(s) where applicable (see Figure 3), completion of the Dermatology Life Quality Index (DLQI) questionnaire and a photograph assessment of the exposed area(s). A standardized set of photographs of the exposed area(s) was taken using the same lighting conditions using Bodystudio ATBM (FotoFinder Systems GmbH, Birnbach, Germany). All photographs were taken with a QPcard 201 attached to the subject's skin for colour correction using QPcolorsoft software, followed by analysis using ImageJ software (NIH, Bethesda, MD).

After this initial assessment, all subjects who presented with PIH were invited to return to our facility for follow‐up assessment of PIH every 6 months. However, insufficient numbers of subjects returned for these follow‐up visits; therefore, these data are not presented.

#### 2.1.2 Statistical analysis

The role of potential risk factors on the occurrence of hyperpigmentation was assessed using a set of patient characteristics and study‐specific variables, which were identified based on clinical considerations and included skin type measured using the Fitzpatrick scale, gender, ethnicity, study enrolment and baseline MED. We then calculated the frequency of subjects with hyperpigmentation and the frequency of subjects without hyperpigmentation in the various risk factor categories.

### 2.2 Study II

#### 2.2.1 Study design

This study was designed to determine the efficacy of using the 2MED UVB irradiation paradigm to induce hyperalgesia, and to assess the prevalence of PIH in subjects exposed to the 2MED UVB paradigm. Each subject visited the clinical research unit for general medical screening and to assess the minimal erythema dose applied to a region of the skin on the subject's back; this visit was performed 3–28 days prior to application of the 2MED UVB paradigm. During the clinical study, 2MED was administered, and hyperalgesia was monitored over the following 36 hr. The subjects then returned to the clinical research unit for two follow‐up visits (6 weeks and 6 months after 2MED exposure), during which the irradiated area was inspected visually and PIH was assessed.

**Figure 2:**
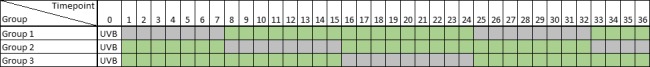
Schematic showing the timing of the PDT measurements obtained in the second study. Eighteen subjects were randomly assigned to three groups. The green boxes indicate PDT measurements

All 18 healthy subjects provided written informed consent. The subjects were divided into three groups containing six subjects each; each group followed a specific measurement protocol in which no measurements were performed for several hours in order to ensure a period of undisturbed sleep (Figure 2). The aim was to minimize the burden placed on the subjects while obtaining the most complete overview of the effects of 2MED UVB with respect to heat pain threshold over time. This design ensured that for each hour after UVB exposure, at least 12 subjects were scored with respect to their heat pain detection threshold (PDT).

#### 2.2.2 Subject selection

After screening and application of the inclusion and exclusion criteria, 18 subjects (nine males and nine females) 18–45 years of age were determined to be eligible and were included in the study. The following exclusion criteria were applied: any current clinically significant medical condition that would have affected sensitivity to pain; history or presence of PIH; the use of concomitant medication (except contraception); dark skin colour (Fitzpatrick skin type IV‐VI); widespread acne, freckles, tattoos and/or scarring on the back; and an MED >355 mJ/cm^2^ at screening.

#### 2.2.3 UVB model

During the screening visit, UVB irradiation was applied using a narrow‐band UVB (TL01) lamp (Philips). To determine the MED, six ascending doses (corresponding to increasing duration of irradiation) were applied to separate 1 cm × 1 cm areas of skin on the subject's upper back. This dosing schedule is based on the average MED of various skin phototypes as reported by Sayre et al. (Sayre, Desrochers, Wilson, & Marlowe, 1981), and ranged from 64 to 1,321 mJ/cm^2^. Twenty‐two to twenty‐six hours after exposure to the six UVB doses, the skin's erythemic response was assessed, and MED was determined visually based on the lowest UVB dose that produced clearly discernible erythema.

**Figure 3:**
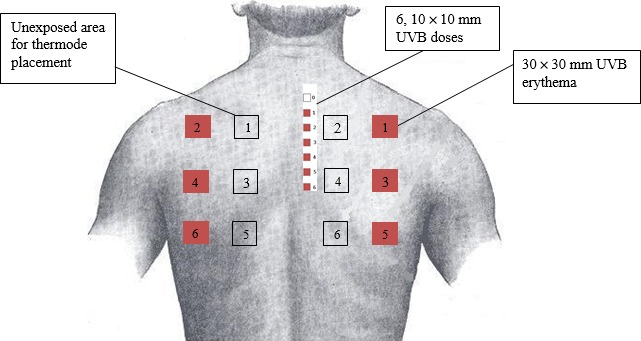
Schematic diagram showing the approximate location of the regions used to determine the MED and the irradiated and non‐irradiated regions used to induce hyperalgesia. To determine the MED, six 1 cm^2^ patches of skin were irradiated at increasing doses. After MED was determined, a separate 3 cm × 3 cm patch of skin was irradiated; a non‐irradiated patch of skin on the contralateral side was used as a control

During the clinical study, two times the individual subject's UVB MED (i.e., 2MED) was applied to the skin over the right scapula prior to the first pain task: the subjects in group 1 received UVB irradiation 8 hr prior to the first pain task, and groups 2 and 3 received UVB irradiation 1 hr prior to the first pain task (see Figure 2). The area of irradiated skin was 3 cm × 3 cm, which matched the dimensions of the thermode used to determine the heat pain threshold (see Figure 3).

Inflammatory pain was assessed first on a patch of control skin contralateral to the site of UVB irradiation, followed by the UVB‐irradiated site. Heat PDT was measured using a 3 cm × 3 cm TSA‐II thermode (Medoc Ltd., Ramat Yishai, Israel). During the test, the thermode temperature started 32°C and increased linearly by 0.5°C/s until the subject reported that the stimulus was first perceived as painful by clicking a mouse button. During each assessment, the average PDT measured using three stimuli was calculated. The schedule for assessing PDT in groups 1, 2 and 3 is shown in Figure 2.

#### 2.2.4 Statistical analysis

Heat PDT was analysed using a mixed‐model analysis of variance with the following fixed factors: treatment (non‐irradiated vs. UVB‐irradiated), time and treatment by time, random factor subject, repeated factor time within subject by treatment with a first‐order autoregressive variance/covariance structure and the pre‐value as covariate. The difference in PDT between non‐irradiated skin and UVB‐irradiated skin was calculated within each time point in the model.

The 2MED and 3MED (extracted from four previous studies at CHDR) heat PDT data were analysed using a mixed‐model analysis of variance using the following fixed factors: treatment (2MED vs. 3MED), time and treatment by time, random factor subject and a repeated factor time within subject with a first‐order autoregressive variance/covariance structure. The difference in PDT between the 2MED and 3MED groups was calculated within the model using the eight post‐UVB time points that were common to both the 2MED and 3MED groups (23, 25, 26, 27, 28, 30, 32 and 34 hr post‐UVB irradiation).

## 3 RESULTS

### 3.1 Study I

Six studies conducted in 2012 to 2015 were included in the analysis, comprising a total of 142 subjects (37 women and 105 men). Five of the male subjects participated in two studies. The characteristics of these 142 subjects are summarized in Table 1.

**Table 1:** Summary of the subjects included in the observational study involving subjects following 3MED UVB exposure

	Total cohort, *N* (%)	Responders, *N* (%)	Responders with PIH, *N* (%)
Subjects	142 (100)	78 (100)	42 (53.8)
Gender			
Female	37 (26.1)	21 (26.9)	11 (52.4)
Male	105 (73.9)	57 (73.1)	21 (54.4)
Ethnicity			
Caucasian	122 (85.9)	67 (85.9)	35 (52.2)
Non‐Caucasian	20 (14.1)	11 (14.1)	0 (0)
Fitzpatrick skin type			
I	2 (1.4)	1 (1.3)	0 (0)
II	25 (17.6)	10 (12.8)	5 (20.0)
III	75 (52.8)	48 (61.5)	28 (58.3)
IV	40 (28.2)	19 (24.4)	9 (47.4)
Time since irradiation (days)			
500–750	25 (17.6)	18 (23.1)	12 (66.7)
751–1,000	69 (48.6)	37 (47.4)	27 (73)
1,001–1,250	39 (27.5)	19 (24.4)	7 (36.8)
>1,751	9 (6.3)	4 (5.1)	0 (0)
MED (mJ/cm^2^)			
251	1 (0.7)	0 (0)	0 (0)
256	1 (0.7)	1 (1.3)	0 (0)
351	4 (2.8)	3 (3.8)	1 (33.3)
355	7 (4.9)	4 (5.1)	1 (25.0)
362	1 (0.7)	0 (0)	0 (0)
467	5 (3.5)	2 (2.6)	1 (50.0)
496	23 (16.2)	13 (16.7)	8 (61.5)
502	9 (63)	2 (2.6)	1 (50.0)
660	17 (12.0)	10 (12.8)	5 (50.0)
702	27 (19.0)	18 (23.1)	11 (61.1)
710	4 (2.8)	2 (2.6)	2 (100)
934	14 (9.9)	8 (10.3)	4 (50.0)
993	22 (15.5)	13 (16.7)	10 (76.9)
1,321	7 (4.9)	2 (2.6)	2 (100)

Note

MED: minimal erythema dose; PIH: postinflammatory hyperpigmentation.

Of the 142 subjects that were contacted, a total of 78 subjects (54.9%) responded; six of these respondents opted to participate from home, and 72 respondents visited our clinic. The mean (SD) age of the respondents was 27.8 (±7.2) years (range: 19–50 years). Forty‐two of the participating subjects (53.8% of respondents) had PIH; the mean age of the participants with PIH was 27.2 (±6.8) years (range: 19–48 years). Table 1 summarizes the prevalence of PIH by ethnicity, gender, MED, Fitzpatrick skin type and time since UVB irradiation. Our analysis revealed that gender, ethnicity and Fitzpatrick skin type were not associated with the prevalence of PIH. However, the remaining study variables were associated with the prevalence of PIH. The prevalence of PIH was the lowest among the subjects in first study group (CHDR0729) and increased with each subsequent study (data not shown). In addition, the MED dose (determined at the initial screening) was generally correlated with the prevalence of PIH.

Overall, the mean total DLQI score among all responding subjects was 2.1 ± 2.8 (range: 0–15). The mean DLQI score for the subjects with PIH was 2.7 ± 3.3 (range: 0–15), and mean score for the subjects without PIH was 1.4 ± 2.0 (range: 0–9). The distribution of DLQI scores among the participants is summarized in Table 2.

**Table 2:** Measurement of dermatology quality of life index

Sum of the DLQI scores	Total responding group		PIH+		PIH−	
	*N*	%	*N*	%	*N*	%
0–1 “no effect at all on patient's life”	49	62.8	23	56.1	23	74.2
2–5 “small effect on patient's life”	20	25.6	11	26.8	6	19.4
6–10 “moderate effect on patient's life”	8	10.3	6	14.6	2	6.8
11–20 “very large effect on patient's life”	1	1.3	1	2.4	0	0
21–30 “extremely large effect on patient's life”	0	0	0	0	0	0

Notes

Calculations made by summing the score of each question resulting in a maximum of 30 and a minimum of 0. The higher the score, the more the quality of life is impaired.

DLQI: Dermatology Quality of Life Index; *N*: number; PIH+: subject with postinflammatory hyperpigmentation; PIH−: subjects without postinflammatory hyperpigmentation.

### 3.2 Study II

A total of 18 subjects (nine males and nine females) completed the study and were included in the final analysis. The mean age of the subjects was 27.1 ± 7.0 years (range: 20–41 years). The characteristics of the subjects in this study are summarized in Table 3.

**Table 3:** Summary of subject characteristics in Study II

Number of subjects	18
Gender	
Female	9
Male	9
Age	
Mean (*SD*)	27.1 (6.8)
Range	20–41
Ethnicity	
White	17
Mixed	1
Fitzpatrick skin type	
II	14
III	4
MED (mJ/cm^2^)	
251	1
351	5
355	12
Weight (kg)	
Mean (*SD*)	74.5 (14.4)
Range	49.4–95.4
Height (cm)	
Mean (*SD*)	176.1 (11.8)
Range	157.9–193.8
BMI	
Mean (*SD*)	23.8 (2.4)
Range	19.6–27.9

Body mass index was defined as weight/(height × 0.01)^2^.

BMI: Body mass index; mJ/cm^2^: millijoule/square centimetre; *SD*: standard deviation.

**Figure 4:**
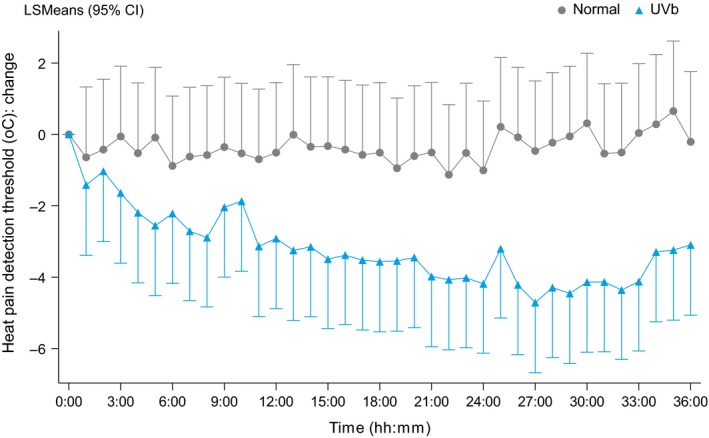
Time course of change in pain detection threshold (PDT) following 2MED UVB exposure. The change in PDT was measured in both the irradiated and non‐irradiated areas and is expressed relative to baseline. The data are expressed as the least square means with 95% CI

Before UVB exposure, the baseline mean PDT on the skin for control (non‐irradiated) and test (irradiated) skin was 44.0 ± 3.6°C and 43.7 ± 4.1°C, respectively. Analysis of the primary endpoint (heat PDT at the irradiated area versus the contralateral non‐irradiated area) revealed a significant difference beginning at 3 hr post‐irradiation (estimate of the difference: 1.58°C, 95% CI: 0.26–2.90, *p *=* *0.0188) onwards; this difference remained significant through the final measurement at 36 hr post‐irradiation. Beginning 13 hr after irradiation, the LSMean estimate of the difference in heat PDT relative to baseline in the irradiated and non‐irradiated skin patches ranged from −2.6°C to −4.45°C (*p *<* *0.0001). Figure 4 shows a time course of the LSMean estimates in the irradiated and non‐irradiated patches.

**Figure 5:**
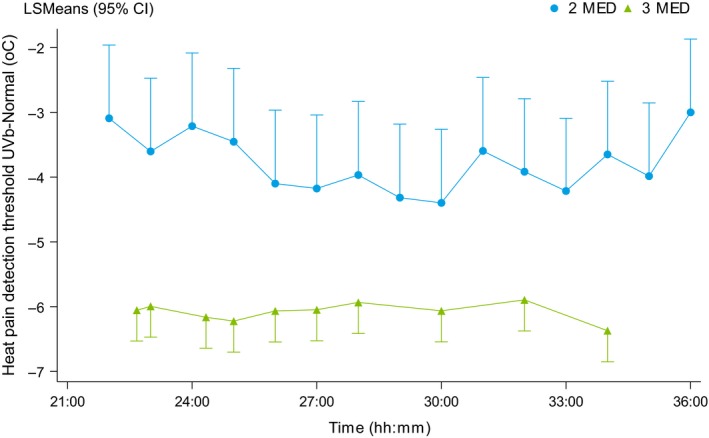
Time course of the difference in PDT between the irradiated area and the control non‐irradiated area following either 2MED or 3MED UVB exposure. The data are expressed as the least square means with 95% CI. 3

The time course for the change in PDT relative to baseline following 2MED and 3MED is presented in Figure 5, which shows that UVB irradiation with 2MED caused in qualitatively similar hyperalgesia compared to 3MED exposure at the same time points; 24–36 hr after irradiation, the average change in PDT following 2MED irradiation was approximately 3–4°C, compared to an average change of approximately 6°C in the 3MED group.

Lastly, we performed a physical examination and examined the photographic data in order to evaluate the incidence of PIH in all 18 subjects both 6 weeks and 6 months after 2MED UVB irradiation. At the 6‐week time point, 11 out of 18 subjects (61.1%) had either minimal (five subjects), mild (four subjects) or moderate (two subjects) hyperpigmentation at the irradiated area. After 6 months, five of the 18 subjects (27.8%) had either minimal (one subject) or mild (four subjects) hyperpigmentation.

## 4 DISCUSSION

This study demonstrates that long‐term effects of the UVB model using the 3MED paradigm pose a major problem for the subjects. Retrospective analysis and assessment of 142 healthy subjects in six previous studies revealed that the prevalence of long‐term PIH in subjects exposed to 3MED UVB for inducing transient hyperalgesia model can be as high as 53.8%. This relative prevalence of PIH may be an overestimation, as patients with PIH may have been more likely to respond to request to participate in a survey of PIH compared to patients without PIH. On the other hand, even our most conservative estimate of nearly 30% is meaningful and should be considered unacceptable for a model that should not have unwanted, long‐lasting and possibly even permanent side effects in healthy subjects.

To the best of our knowledge, such a high prevalence of long‐term UVB‐induced PIH has not been reported previously. One study that evaluated long‐lasting molecular changes in the skin of subjects after repetitive UV irradiation reported one subject who developed hyperpigmentation 520 days after repetitive, cumulative UV exposure (Brenner et al., 2009). The high prevalence in our cohort, combined with the paucity of reports regarding UVB‐induced PIH, suggests the possible under‐reporting of long‐lasting side effects in healthy subjects. The significant and widespread under‐reporting of adverse drug reactions is a well‐known phenomenon (Hazell & Shakir, 2006); in contrast, virtually no data are available with respect to adverse reactions associated with the UVB inflammation model. Several factors may contribute to this phenomenon. First, an assessment of harm by clinicians may not necessarily represent the subjects’ experience. Second, even if harm is detected, it may not be reported appropriately by the investigators, or its reporting may be influenced by the study sponsors, particularly in the case of commercial sponsors. Finally, short‐term follow‐up might not be adequate to detect potential long‐term side effects (Seruga et al., 2016). This last fact may be particularly relevant here. The under‐reporting of long‐term side effects can prevent researchers from learning from these incidents in order to improve both the safety of study subjects and the design of future studies.

UVB‐induced inflammation and PIH are complex processes involving both molecular and cellular changes that lead to the overproduction of melanin and/or the irregular dispersion of pigment following inflammation. Mechanistically, the release of prostanoids, cytokines, chemokines and other inflammatory factors is stimulated in both UVB‐induced inflammation and PIH (Davis & Callender, 2010). Moreover, several studies found the leukotrienes C4 and D4, prostaglandin E2, prostaglandin D2, thromboxane‐2, interleukin‐1 (IL‐1), IL‐6, tumour necrosis factor alpha (TNF‐α), epidermal growth factor and reactive oxygen species such as nitric oxide (Chang, 2009; Ortonne, 1992; Taylor et al., 2009; Tomita, Maeda, & Tagami, 1992) have melanocyte‐stimulating properties. Pro‐inflammatory cytokines such as IL‐1 also increase the expression of bradykinin, a potent algogenic compound that is produced following tissue injury and may mediate UVB‐induced hyperalgesia and pain (Eisenbarth, Rukwied, Petersen, & Schmelz, 2004; McMahon, Bennett, & Bevan, 2006). Bradykinin‐induced pain and erythema are mediated in UVB‐inflamed skin, possibly via an up‐regulation or de novo expression of receptor proteins (Eisenbarth et al., 2004; Perkins & Kelly, 1993). For example, the bradykinin receptor is sensitized by inflammatory mediators, particularly prostaglandins (Poole, Lorenzetti, Cunha, Cunha, & Ferreira, 1999; Tonussi & Ferreira, 1997), which are produced in UVB‐inflamed skin (Clydesdale, Dandie, & Muller, 2001; Soter, 1990), ultimately causing the sensitization of cutaneous nociceptors (Liang, Haake, & Reeh, 2001; Petho, Derow, & Reeh, 2001). Furthermore, Andersen, Abrams, and Maibach, (1992) systematically examined the correlation between an UVB dose and inflammation; however, some details regarding the underlying mechanism are still unknown, and the notion that a higher UVB dose increases the molecular and/or cellular changes in the dermis that lead to PIH warrants further investigation.

Although most of the subjects with PIH in the observational study reported that their daily life was not severely affected by the hyperpigmented area(s), many reported that several aspects were affected, including self‐consciousness, social well‐being and interpersonal relationships. Nevertheless, based on the DLQI scores, the PIH had less of an effect compared to other hyperpigmentation‐related disorders (Maymone et al., 2017). Although analysis of possible risk factors for hyperpigmentation, including ethnicity, skin colour and heat hyperalgesia, did not reveal a clear correlation between these factors and the prevalence of PIH, it was found that generally speaking subjects with a high MED had a higher risk of PIH; in addition, the prevalence of PIH generally decreased with increasing time following UVB exposure. Several epidemiological studies found that PIH tends to occur more frequently among dark‐skinned individuals compared to individuals with lighter skin tones (Alexis, Sergay, & Taylor, 2007; Chua‐Ty, Goh, & Koh, 1992; Halder, Grimes, McLaurin, Kress, & Kenney, 1983). This study could not confirm these results, as ethnicity and Fitzpatrick skin type were not distributed evenly among the subjects.

In study II, subjects were excluded with a MED score >355 mJ/cm^2^ and a Fitzpatrick skin type IV. The MED was multiplied by twofold instead of a threefold in order to try to keep the occurrence of PIH to a minimum. No cases of PIH have been described in literature when using the 2MED UVB model. Validation of the 2MED UVB model confers hyperalgesia is consistent with previous studies using the same paradigm (Bishop, Ballard, Holmes, Young, & McMahon, 2009; Ing Lorenzini et al., 2011, 2012; Rother & Rother, 2011). We also found that 2MED UVB model induced primary hyperalgesia as early as 3 hr after irradiation, and this response was relatively stable for up to 36 hr after irradiation.

Compared to 3MED UVB, 2MED UVB caused slightly less pronounced hyperalgesia, which was reflected by a difference in average PDT of approximately 2°C between 2MED and 3MED; this finding is consistent with previous reports of dose‐dependent sensitization of cutaneous nociceptors (Benrath, Gillardon, & Zimmermann, 2001; Bishop et al., 2009; Gustorff et al., 2013; Harrison et al., 2004). However, the relatively high prevalence of long‐term PIH in the 3MED cohort indicates that the 3MED model should be used with caution, as its use can lead to a negative risk–benefit balance. Nevertheless, the UVB inflammation model is an established model for inducing cutaneous hyperalgesia, making it valuable for use in studies designed to investigate the investigating effects of analgesics in the setting of hyperalgesia (Loudon et al., 2018; Okkerse et al., 2017; Sycha et al., 2003; van Amerongen et al., 2018). Importantly, this model provides a consistent level of efficacy with low inter‐subject variability (unpublished data) and high test–retest reliability (Mørch, Gazerani, Nielsen, & Arendt‐Nielsen, 2013). Given these advantages, the UVB model is also considered suitable for modelling inflammatory pain and is therefore used to measure the effects of non‐steroidal anti‐inflammatory drugs (NSAIDs; Bishop et al., 2009; van Amerongen et al., 2016).

Because of the study design, the effect of analgesics on hyperalgesia induced by 2MED UVB exposure was not examined. Thus, an important question that remains to be addressed is whether or not the slightly lower hyperalgesia induced by 2MED is still sufficient to test the efficacy of analgesic compounds. To date, one study used the 2MED paradigm and was able to demonstrate the efficacy of a combination of paracetamol and ketorolac in reducing hyperalgesia (Ing Lorenzini et al., 2011). Finally, one of the main reasons to execute this study was to develop a valid pain model with minimal risk of hyperpigmentation. The prevalence of PIH 6 months after exposure to 2MED is similar to the prevalence in the 3MED group after a follow‐up between 901 and 1,128 days. As the prevalence of PIH among the subjects exposed to 3MED declines over time as demonstrated by the lower prevalence of PIH among subjects with a longer time since exposure than among patients with a shorter time since exposure (see Table 1), it is to be expected that PIH will fall well below 20%. To test this hypothesis, follow‐up will continue for subjects in whom hyperpigmentation was still present 6 months after exposure.

In conclusion, our observational study revealed that long‐lasting PIH is relatively common among healthy subjects previously exposed to 3MED UVB irradiation, providing the first report of this adverse side effect in association with this model. Given that 2MED UVB is associated with a reduced prevalence of PIH yet still produces stable hyperalgesia, this model should be tested for use in evaluating the efficacy of analgesics in the early stages of development.

## CONFLICT OF INTEREST

None to declare.

## AUTHOR CONTRIBUTION

All authors made substantial contributions to the development and design of the protocol; to the acquisition, analysis and interpretation of the data; and to the writing and/or revising of the manuscript. All authors approved the final submitted version for publication.
